# ELISA Methods Based on Monoclonal Antibodies for the Serological Diagnosis of Lumpy Skin Disease

**DOI:** 10.1155/2023/8378153

**Published:** 2023-07-27

**Authors:** Stefano Baselli, Giulia Pezzoni, Marcella Sabino, Santina Grazioli, Janika Wolff, Bernd Hoffmann, Valentin Shtjefni, Lorenzo Capucci, Emiliana Brocchi

**Affiliations:** ^1^Istituto Zooprofilattico Sperimentale della Lombardia e dell'Emilia Romagna (IZSLER), via Bianchi 9, 24125, Brescia, Italy; ^2^Institute of Diagnostic Virology, Federal Research Institute for Animal Health, Friedrich-Loeffler-Institut, Südufer 10 D-17493, Greifswald-Insel Riems, Germany; ^3^Animal Health Department, Food Safety and Veterinary Institute, “Aleksander Moisiu” No 82, Tirana 1005, Albania

## Abstract

Lumpy skin disease (LSD) is a notifiable, transboundary cattle disease that spreads rapidly and has a relevant economic impact. The etiological agent is the LSD virus (LSDV), genus *Capripoxvirus*, and family *Poxviridae*. To date, LSDV is widely present in Africa, Asia, and in transcontinental regions like Russia, Turkey, and the Middle East, thus representing a continuous threat to free neighbours. Appropriate serosurveillance programs can complement disease control, inform about its spread, and enable the assessment of vaccination campaigns. Since reliable and practical diagnostic tools could improve serological surveillance, this study aimed to produce and characterize monoclonal antibodies (MAbs) that allowed us to develop ELISA tests for the serological detection of LSD. Four MAbs recognizing a 35 kDa viral protein were selected and used to develop and optimize competitive and indirect ELISAs. Both assays detected seroconversion within 14 days postinfection (dpi) in 18 cattle experimentally infected with LSDV and sequentially sampled for up to 4 weeks. The two novel ELISAs detected also antibodies raised by other capripoxviruses: as observed in cattle, both assays revealed seroconversion within 14 dpi in all nine sheep experimentally infected with sheeppox virus (SPPV), while in eight goats infected with goatpox virus (GTPV) competitive ELISA identified seropositivity earlier and in more animals compared to indirect ELISA. Overall, the sensitivity performance of both developed ELISAs resulted comparatively superior to those of virus neutralization test and the commercial Id.Vet ELISA. Testing of about 200 negative sera from each species recorded a single false-positive cattle in the indirect ELISA, which gave a specificity of 99.5%, whereas for the competitive ELISA, the diagnostic specificity was 100% irrespective of the species tested. The results enable concluding that both new tests correctly detect anti-LSDV antibodies in cattle and can also be reliable tools to recognize antibodies to SPPV and GTPV.

## 1. Introduction

Lumpy skin disease (LSD) is an infectious viral disease that primarily affects cattle. It could be asymptomatic (generally, 10%–40% of cattle show subclinical course), and it depends on different factors (*i.e*., host immune status, virus strain, and breed). LSD clinical signs include fever, skin nodules, lesions of the oral mucous membranes, of the respiratory, and digestive tract. This disease is characterized by low-mortality rate, often less than 10% of infected animals die, but could reach up to 85%, and the morbidity rate usually is between 1% and 10% reaching up to 75% [1]. The World Organization for Animal Health (WOAH) categorizes LSD as a notifiable disease due to the significant economic impact of possible outbreaks. The etiological agent is the Lumpy skin disease virus (LSDV), a large double-stranded DNA virus with an oval or brick-shaped morphology, and a genome of about 150 kDa. LSDV belongs to the *Capripoxvirus* genus, *Poxviridae* family, which also includes the Sheeppox virus (SPPV) and the Goatpox virus (GTPV), all sharing a sequence homology greater than 90% [2]. Among the four reported virion forms, two of them can infect the host: the intracellular mature virus (IMV) and the extracellular enveloped virus (EEV), with EEV surrounded by an additional membrane containing several virus proteins [3]. LSDV spreads over long distances mainly through an indirect process by blood-feeding arthropod vectors such as mosquitoes, biting flies, and ticks [4, 5].

LSD was first reported in 1929 in Zambia and from there it disseminated throughout the African continent. The first entry to Europe was in 2015 in Greece, then it rapidly spread to the Balkans counties [6]. Only an intense coordinated vaccination campaign in some of the affected states allowed control of the diffusion a few years after LSD onset [7]. Over the last 4 years, LSD has not been reported in other European countries, but it has been diagnosed in Turkey and Russia, and has spread across much of Southern Asia [8–10]. To avoid the epizootic onset, preventive and containment measures are necessary. An effective eradication campaign includes a good and timely diagnosis of the disease using suitable tools. End-point PCR, real-time PCR, and virus isolation are effective methods that identify the virus at the early stage of infection [11]. Other methods addressed at the detection of antibodies, like virus neutralization test (VNT), enzyme-linked immunosorbent assay (ELISA), immunoperoxidase monolayer assay (IPMA), immunofluorescence, and western blotting (WB), can detect the host immune response at later stages of infection when seroconversion has occurred [12]. Although this infection principally triggers a cell-mediated response, the humoral response also plays an important role in protecting the host, allowing the detection of infected animals [13]. LSDV carries several highly immunogenic proteins, such as those located in the envelope, leading to a good humoral response [14, 15]. The reference serological method detecting the presence of neutralizing antibodies is the VNT, which is however labor intensive, requiring maintenance of cell culture and the manipulation of live virus. Among other serological tests, ELISA is the most useful method for field surveillance since it does not require specific equipment, it is fast and it permits to analyze a large number of samples at a reasonable cost [16]. To date, ID Screen Capripox Double antigen (DA) ELISA (ID.vet, Grabels, France), based on a capripox recombinant antigen, is the only commercial ELISA kit available for the detection of antibodies against capripoxvirus [17, 18]. Indirect ELISA assays using recombinant proteins have been also described by some authors [19–22], as well as recombinant antigens have been used to study capripoxviruses immunogenicity [23]. An indirect ELISA assay using the inactivated purified SPPV has been also described by Babiuk et al. [24], however, the viral purification procedure was complicated, requiring ultracentrifugation of the viral solution through a sucrose cushion followed by a continuous sucrose gradient. To our knowledge, no ELISA assay for antibody detection, which uses monoclonal antibodies (MAbs) has been described so far. This work aimed to produce and characterize MAbs against LSDV, and use them to develop two different ELISAs for antibody detection in LSDV exposed animals. These assays were based on two different principles, namely competitive and indirect ELISA. Increasing the spectrum of serological tests for LSDV with these novel assays, which are also suited to be easily made in the format of ready-to-use kits, opens further opportunities to improve LSD surveillance and control, at the same time making available additional tools for the study of the immune response to LSDV.

## 2. Methods

### 2.1. Viral Antigen Production and Inactivation

The WOAH Reference Laboratory for LSDV (Pirbright Institute, UK) gently provided the LSDV Neethling strain, which was amplified on primary cell cultures of ovine testis (TSO) to produce the viral stock. Subsequently, OA3.Ts (*Ovis aries testis*) cell line (ATCC CRL-6546) was used to propagate the virus and obtain the viral antigen, as they are easier to propagate, compared to primary cells, and produce a good viral titer [25, 26]. At the maximum cytopathic effect, infected OA3.Ts cells underwent a freeze-thawing cycle. The crude viral suspension was collected, clarified by centrifugation, filtered through 0,45 *µ*m filters, and the virus was inactivated with *β*-propiolactone 0.01%, which is used to inactivate viruses while maintaining the virus external structure unchanged [27]. This inactivated LSDV preparation was used as antigen in the ELISA tests developed for antibody detection. To produce and characterize MAbs, an inactivated LSDV (1 L) underwent also a purification procedure by differential centrifugation. In brief, a first centrifugation step was performed for 1 hr at 11,000 *g* and the pellet was resuspended to 200x in Tris 10 mM pH 8.0 buffer; then, the viral solution was homogenized, sonicated, and further centrifuged for 15 min at 1,000 *g*. The supernatant was collected, and the pellet was treated for a second step of solubilization as previously described. All the supernatant collected was ultracentrifuged through a 36% w/v sucrose cushion at 18,000 rpm for 90 min at 4°C using an SW40 rotor (Beckman Coulter). The pellet obtained was resuspended in 2 mL of Tris 10 mM pH 8.0 buffer with a final concentration of 500×. The final purification step consisted of ultracentrifugation through a 24%–40% continuous sucrose gradient at 14,000 rpm in SW40 rotor for 40 min at 4°C (Beckman Coulter) followed by collection of 1.5 mL gradient fractions. Three central opalescent fractions were mixed, diluted to 10 mL with Tris 10 mM pH 8.0 buffer and pelleted by ultracentrifugation at 18,000 rpm for 90 min at 4°C using SW40 rotor; the pellet was resuspended in 2 mL of Tris 10 mM pH 8.0 buffer (final concentration 500×). The presence of the virus during the subsequent purification steps was monitored by electron microscopy; the protein concentration in the purified viral preparation was 1 mg/ml according to the BCA assay (Pierce™ BCA Protein Assay Kit, Thermo Fisher Scientific, Waltham, MA, USA).

### 2.2. Sera Collection

Experimental sera were obtained from infections carried out at Friedrich Loeffler Institut (Greifswald-Insel Riems, Germany). In particular: 90 cattle sera derived from 18 animals [28] of which six inoculated with the LSDV Neethling attenuated vaccine strain, eight infected with the LSDV strain circulating in North Macedonia in 2016, and four infected with a LSDV Nigerian strain isolated in 2019 [29] from each cattle, sera were collected before infection and then weekly up to 28 days postinfection (dpi), as detailed in Table S1. Twenty-eight goat sera were obtained from eight goats, of which six experimentally infected with the GTPV strain named GTPV-“V/103” (GenBank accession number MW020570) and two in-contact, as detailed earlier [30]. Sera were sampled at different time-points up to 28 dpi or, for those goats euthanized earlier due to severe clinical signs, only up to the first- or second-week pi (Table 1). Thirty-three sheep sera were collected during the experimental infection previously described [31], where two groups of six sheep each were, respectively, infected with SPPV-“India/2013/Surankote” (GenBank accession number MW167070) and SPPV-“Egypt/2018” (GenBank accession number MW167071), and were sampled weekly up to 28 dpi. Sera accessible for the present study derived from nine of those sheep, six of them available only up to 14 dpi (Table 2). Furthermore, a total of 192 cattle sera, 187 goat sera, and 241 sheep sera were collected in Italy (a capripoxvirus-free country) and used as negative samples for the definition of the cutoff of the tests. Cattle sera were collected in 27 livestock in the Lombardy region, in Northern Italy; goat and sheep sera were collected in 28 and 18 livestock, respectively, in Northern Italy (Lombardy and Emilia-Romagna regions).

### 2.3. Monoclonal Antibodies Production

The MAbs produced are the result of two immunizations of BALB/c mice carried out, respectively, with the purified (obtained by ultracentrifugation through sucrose gradient) or the semipurified LSDV (obtained by ultracentrifugation through sucrose cushion). BALB/c mice (one mouse for each of the above antigen preparation) were primed subcutaneously with the LSDV in Freund's complete adjuvant and boosted intraperitoneally with the same antigen dose in phosphate-buffered saline (PBS) 50 days later. A second booster was performed 1 month later by intraperitoneal injection of the antigen at the same conditions as the previous one. Three days later mice were euthanized and splenocytes were fuzed with NS0 myeloma fusion cells. Cell fusion and cloning of positive hybridomas were performed following procedures standardized in our laboratory [32]. The hybridomas were screened by indirect ELISA for the secretion of the LSDV specific antibodies against the homologous virus used for the immunization and uninfected OA3.Ts cell lysates. Following initial characterization, selected hybridomas were grown in Integra CELLine flasks (Merck Life Science, Darmstadt, Germany) for at least 2 months, with two to three weekly harvests of culture medium containing MAb concentrations in the range 0.2–1.5 mg/mL. Four MAbs were purified from exhausted culture media using protein A [33] and conjugated with Horseradish Peroxidase (HRP, Merck Life Science, Darmstadt, Germany) according to the protocol previously described [34].

### 2.4. Monoclonal Antibodies Characterization

For the MAbs characterization, we used methodologies previously developed in our laboratory and adapted to the different antigens under study [32, 35, 36].

#### 2.4.1. Indirect ELISA

A saturating concentration of purified LSDV antigen in carbonate/bicarbonate buffer (pH 9.6) was adsorbed onto microplates (NUNC, Maxisorp, Roskilde, Denmark) by overnight incubation at 4°C. After three washing cycles with PBS-Tween buffer (PBS with 0.05% Tween 20), supernatants of hybridomas cultures were incubated for 1 hr at 37°C. After three washes with PBS-Tween buffer, incubation with a secondary antibody (peroxidase-conjugated rabbit IgG antimouse immunoglobulins, homemade) was carried out. After three final washes, 0.5 mg/mL of OPD (o-phenylenediamine) diluted in phosphate–citrate buffer (pH 5.6) and supplemented with 0.02% H_2_O_2_ was distributed and incubated for 10 min at room temperature; the reaction was stopped with H_2_SO_4_ (1 M). Absorbance values were read at 492 nm wavelength using an ELISA reader (Multiscan Ascent spectrophotometer, Thermo Fisher Scientific, Waltham, MA, USA). The reagents dilution buffer consisted of PBS buffer (pH 7.4) containing 0.05% Tween 20 and 1% yeast extract. About 50 *µ*L per well of reagent was used in each step.

#### 2.4.2. Western Blotting (WB)

WB was performed using a standard protocol [37]. In brief, the semipurified LSDV was denatured in SDS (sodium dodecyl sulfate) and *β*-mercaptoethanol; the denaturated LSDV proteins were separated on precast polyacrylamide gels at 4%–12% gradient concentration (Novex® Tris-Glycine, Thermo Fisher Scientific, Waltham, MA, USA) and then transferred to polyvinylidene difluoride (PVDF) membranes (Hybond®-LFP polyvinylidene difluoride, Cytiva Amersham, Little Chalfont, Amersham HP7 9NA, UK) [38]. Supernatants of hybridoma cultures containing MAbs were diluted 1 : 5 in a PBS pH 7.2 containing 1% w/v of bovine albumin and 0.05% v/v of Tween20. MAb binding was detected by incubation with a homemade peroxidase-conjugated rabbit anti-mouse IgG as secondary antibody, followed by the chromogenic substrate Tetramethylbenzidine (TMB) (Novex™ HRP Chromogenic Substrate, Thermo Fisher Scientific, Waltham, MA, USA).

Western blotting was carried out as described above with LSDV positive- and negative-cattle sera that were diluted 1 : 20, while the antibody binding was detected with an HRP-conjugated anti-ruminant IgG MAb (produced from a proprietary hybridoma).

#### 2.4.3. Competitive Binding Assays

Competitive ELISAs were designed to analyze the capability of anti-LSDV antibodies raised in sera of infected cattle to inhibit the binding of MAbs to the homologous antigen. About 50 *µ*L of known positive cattle sera, at sequential dilutions, were incubated for 1 hr at 37°C in immunoplates coated with the purified LSDV, together with 25 *µ*L of a predetermined dilution of hybridoma supernatant which had given an absorbance value of 1.5 ± 0.5 in a preliminary titration. The binding of the MAb was detected with a secondary antibody (antimouse immunoglobulins peroxidase conjugated) and the colorimetric reaction was achieved as described above for indirect ELISA. When the epitope target of the MAb is also recognized by positive sera, the blocking of the epitope by serum antibodies inhibits color development. A competitive ELISA was also designed to evaluate the reciprocal competition between those MAbs recognizing the same protein in WB. Here, the previous protocol was sligthly modified. In detail, the inactivated LSDV (crude lysate, supernatant of infected cells) was immunocaptured into the ELISA microplate by a capture MAb preadsorbed at 2 *µ*g/mL in carbonate/bicarbonate buffer (pH 9.6); capture MAbs 2F10 or 2C6 were used, on the basis of the results of sandwich ELISA (described below). After plates washing, sequential dilution of hybridomas culture medium expressing each MAb were incubated for 1 hr at 37°C, and the competitor peroxidase-conjugated MAb was added and incubated for a further 1 hr at 37°C. The reagents dilution buffer consisted of PBS buffer (pH 7.4) containing 0.05% Tween 20 and 1% yeast extract the inactivated LSDV After three further washes, a colourimetric reaction was developed as described above. About 50 *µ*L per well of reagent was used in each step.

#### 2.4.4. MAbs Isotype Identification

The MAbs isotype was determined through a homemade ELISA using anti-IgG and IgM isotypes specific reagents (Merck Life Science, Darmstadt, Germany). In brief, the purified LSDV was absorbed to the plate wells; then, hybridomas cultures supernatants were incubated for 1 hr at 37°C. Each MAb was subsequently incubated for 1 hr at 37°C with purified goat immunoglobulins against a single isotype of mouse immunoglobulins (Merck Life Science, Darmstadt, Germany) Finally, a peroxidase-conjugated monoclonal antibody antigoat IgG was delivered, and after 1 hr of incubation, the colorimetric reaction was developed and read as described above. The reagents dilution buffer consisted of PBS buffer (pH 7.4) containing 0.05% Tween 20 and 1% yeast extract. About 50 *µ*L per well of reagent was used in each step; three washing steps with PBS-Tween 20 were performed after each incubation.

#### 2.4.5. MAbs Sandwich ELISA

Sandwich ELISAs were designed as the basis for the development of a competitive ELISA for anti-LSDV antibody detection. Four anti-LSDV MAbs were selected for this study; for the function of antigen-capture antibody each MAb was adsorbed to NUNC plate at a saturating concentration of 2 *µ*g/mL in a carbonate/bicarbonate buffer (pH 9.6) by overnight incubation at 4°C. Sequential dilutions of the antigen, in the format of inactivated LSDV crude lysate, were then incubated for 1 hr at 37°C, followed by serial dilutions of each of the four anti-LSDV MAb peroxidase-conjugated, for checkerboard titration of immune-reagents. After 1 hr incubation at 37°C, the colourimetric reaction was developed. Three washings with PBS-Tween 20 were performed after each incubation; 50 *µ*L per well of reagent was used in each step; reagents dilution buffer and development of the chromogenic reaction were as described for all previous ELISAs.

### 2.5. Competitive ELISA for anti-LSDV Antibody Detection

To develop the competitive ELISA, the optimal dilutions of the viral antigen and the chosen peroxidase-conjugated MAb were preliminarily determined by a checkerboard titration, providing a spectrophotometric reading of 1.5 ± 0.5 OD (optical density) at a wavelength of 492 nm. The test was carried out using the inactivated crude LSDV immune-captured into NUNC Maxisorp plate wells using the 2F10 MAb, coated as described in the sandwich ELISA (2 *µ*g/mL) as capture antibody. After plate washing with PBS-Tween to remove unbound antigen, serum samples and positive and negative control sera diluted 1 : 5 were incubated 1 hr at 37°C with the trapped antigen, then 25 *µ*L of 2C6 peroxidase-conjugated MAb was supplied for a further 1 hr incubation. After three PBS-Tween washing steps to remove any unbound material, the OPD substrate was delivered as previously described. The reaction was stopped after 10 min at room temperature with 50 *µ*L of H_2_SO_4_ (1 M). Dilution buffer for antigen, sera and conjugated MAb was composed of PBS (pH 7.4) containing 1% yeast extract and 0.05% Tween 20. About 50 *µ*L/well were used in each step. The plates were analyzed using a Multiscan Ascent spectrophotometer at 492 nm wavelength. Results were expressed as percentage of inhibition (PI) of each serum sample compared to the peroxidase-conjugated MAb control reaction, according to the following formula:

(1) $$\begin{array}{c} PI=100-\frac{Test\;serum\;OD\;Value}{OD\;Value\;of\;test\;negative\;serum}\times 100. \end{array}$$

### 2.6. Indirect ELISA for anti-LSDV Antibody Detection

Microtiter plates (NUNC, Maxisorp, Roskilde, Denmark) were coated with an oversaturing concentration of the purified 2F10 MAb (3 *µ*g/mL) and incubated overnight at 4°C. Three PBS-Tween washing were performed, and the inactivated crude LSDV was dispensed at saturating dilution in odd rows, while only dilution buffer (same components described above) was supplied to even rows. After 1 hr at 37°C and three washes with PBS-Tween, sera diluted 1 : 50 were dispensed in duplicate (both in the well with and without antigen). Then, 1 hr incubation at 37°C and three washes with PBS-Tween buffer were carried out. A proprietary anti-ruminant IgG MAb (named 1G10) conjugated with peroxidase was provided to each well and incubated for 1 hr at 37°C. After three final washes, the OPD substrate was added, and the reaction was stopped as previously described. Plates were analyzed using a Multiscan Ascent spectrophotometer at 492 nm wavelength. The inactivated LSDV, sera and peroxidase-conjugated antibody were diluted in PBS-Tween buffer with 1% yeast extract. To determine the results, the net OD value was calculated by subtracting the OD value of the well without antigen from the OD value of the well containing the antigen.

### 2.7. Statistical Analysis

The correlation between competitive and indirect ELISA tests was calculated with Pearson coefficient, which is widely used to quantify the association of two variables among samples [39, 40].

## 3. Results

### 3.1. Selection of MAbs to Design Serological ELISAs

The characterization of MAbs was essentially addressed at the selection of best suited MAbs to the development of ELISAs for the detection of anti-LSDV antibodies. Out of 75 MAbs specifically reacting with the purified LSDV antigen used for mice immunization and negative against uninfected OA3.Ts cells cryolysate, 14 MAbs (Table 3) recognized a 35 kDa protein in the WB assay; the same band was also the one best efficiently recognized by sera of LSDV infected cattle (not shown), confirming the strong immunogenicity of this protein. Therefore, further characterization was focused on these MAbs, all resulting of IgG1 or IgG2a isotype and all providing a strong signal (OD values) in the indirect ELISA used for hybridomas screening (Table 3).

Since the capability of a MAb to compete with positive sera is a prerequisite for its use in the development of a serological competitive ELISA, the four best performant MAbs in the inhibition of the binding of LSDV positive cattle sera to the virus, according to the results of preliminary competitive binding assays, were then selected for following uses. To this purpose, these four MAbs, named 3H5, 2C6, 2F10, and 2F12, were purified and conjugated to peroxidase. This also enabled us to perform reciprocal competitive ELISA assays between MAbs directed against the 35 kDa viral protein, in order to investigate on the existence of possible relationships between the respective target epitopes. Only three out of four conjugated MAbs were used in reciprocal competitive studies because one MAb (2F12) lost part of its reactivity after conjugation, probably due to steric hindrance caused by the peroxidase molecule. The competitive profiles (Table 3) of the four selected MAbs showed different patterns, suggesting that they may identify different epitopes along the protein: (i) 2C6 and 3H5 MAbs completely competed each other with reciprocal percentage inhibition over 90%, which is an indication that both MAbs bind to a similar position of the target viral protein, and were not significantly inhibited by any other MAb; (ii) 2F10 MAb competed poorly with 3H5 and 2C6 and showed a profile similar to several other MAbs; interestingly, it was broadly inhibited by most MAbs, though to a different extent; (iii) 2F12 MAb displayed a profile similar to 2F10, though with a reduced strength of competition, that could indicate a different affinity for the same epitope, or even different target epitopes.

### 3.2. ELISA Tests Development

**Figure 1:**
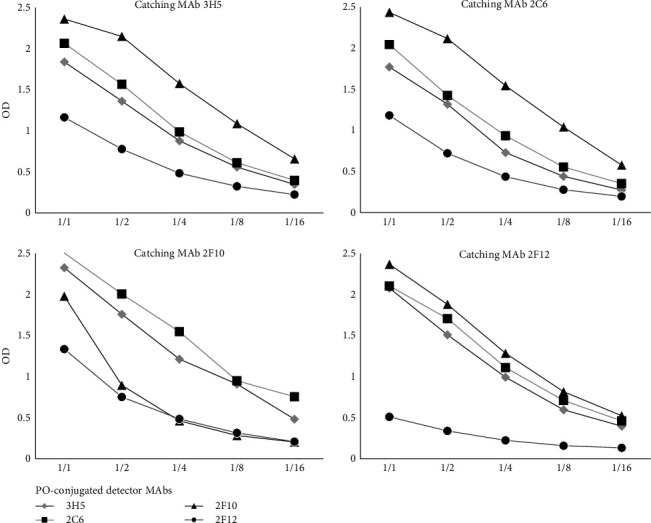
Sandwich ELISA performed with all combinations of MAbs used as capture and detector antibody (peroxidase-conjugated MAb). LSD crude viral antigen was diluted and tested (shown in the *x*-axis); on the *y*-axis, the recorded value of optical density (OD) is indicated.

One of the essential requirements for an easy production of serological assays is the possibility to use nonpurified and nonconcentrated antigens. In order to investigate this feasibility, the capability of the four selected MAbs used as coating antibody, to capture and immune purify the LSDV antigen from a crude virus preparation was analyzed by means of a sandwich ELISA. All the combinations of the four MAbs, used either as capture or conjugated (detector) antibodies, were analyzed; it is noteworthy that the sandwich ELISA, which is essentially a test for antigen detection, may also represent the basis for the development of a serological competitive ELISA, where positive sera hinder the binding of the conjugated MAb, thus inhibiting the development of the reaction. The sandwich ELISA results highlighted a similar reactivity profile for 3H5 and 2C6 MAbs:, both worked efficiently either as coating Ab (LSDV capture MAbs) and as peroxidase-conjugated antibody (detector MAbs), and both presented a strong signal when combined with the 2F10 detector MAb, which identified a different epitope, according to the reciprocal competition profiles. 2F10 MAb was the most efficient detector antibody, except when it was used in a homologous combination (both catching and detector antibody). 2F12 MAb captured the LSDV antigen when used as coating antibody, but conserved poor reactivity when conjugated with peroxidase (PO) (Figure 1). Three antibody pairs (2F10/3H5-PO, 2F10/2C6-PO, and 2C6/2F10-PO) were selected as candidates for the competitive ELISA development; to find out which performed better, they were evaluated with a small panel of LSDV positive cattle sera (gently provided by the EU Reference Laboratory for diseases caused by capripoxviruses, SCIESANO, Belgium) and with 192 negative bovine sera. Positive sera efficiently inhibited the binding of any of the three conjugated MAbs; however, the distribution of negative sera was more homogeneous and specific when 2C6 was used as conjugated MAb (data not shown). As a result, 2F10 and 2C6 peroxidase-conjugated were chosen as the best match of capture and detector antibodies, respectively, to design the serological ELISA based on the competitive principle. For the feasibility study of an indirect ELISA, a preliminary evaluation of the four MAbs as antigen capture antibody showed that all of them correctly captured the inactivated LSDV from a crude preparation and presented it to the recognition by positive sera (not shown). 2F10 MAb was selected as capture MAb, in order to handle a single capture MAb for both ELISAs.

### 3.3. Evaluation of Cattle Sera in the Competitive and Indirect ELISAs

**Figure 2:**
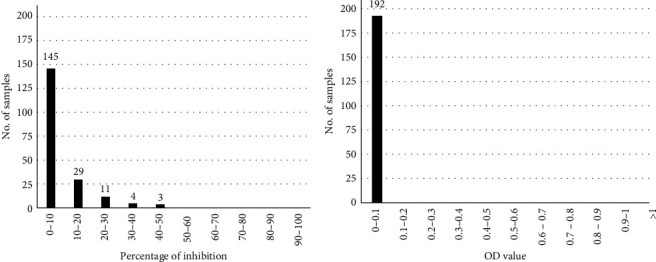
Values distribution obtained examining a population of 192 LSD-negative cattle sera. Results are expressed as percentage of inhibition in the competitive ELISA (a) and as optical density (OD) in the indirect ELISA (b), and the values are reported on the *x*-axis. On the *y*-axis, the number of analyzed samples is shown.

The evaluation of 192 LSDV negative cattle sera in competitive ELISA showed a PI distribution included in a range of 0%–50% PI, with 90% of them confined in the range 0%–20% PI (Figure 2(a)). The cutoff value of 50% PI was therefore established for the competitive ELISA, resulting in 100% test specificity. In the indirect ELISA all the 192 LSDV negative sera presented net OD values lower than 0.1 OD (Figure 2(b)); however, considering also the reactivity profile of sera collected from the 18 cattle before the experimental infection, the cutoff value was fixed at 0.2 OD with a specificity of 99.5% (1 false-positive out of 210 negative samples).

**Figure 3:**
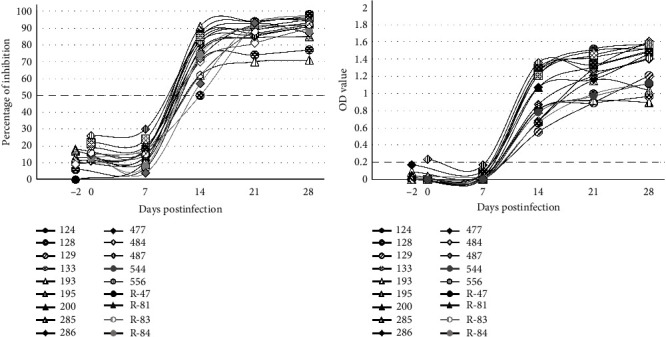
Seroconversion detected in cattle sera collected at different time points after experimental infection with three LSDV strains. Six cattle were infected with the vaccine strain Neethling, eight with a 2016 isolate from North Macedonia, and four with a 2019 isolate from Nigeria. Sera were tested in competitive ELISA (a) and indirect ELISA (b). On the *x*-axis, dpi are shown, while on the *y*-axis the percentage of inhibition (a) and the optical density (OD) value (b) are reported. The dashed line indicates the cutoff value for both analyses.

The test of sera of LSDV experimentally infected cattle sequentially collected up to 28 dpi showed that both ELISAs detected a clear seroconversion from 7 to 14 dpi (Figures 3(a) and 3(b)). Exceeding 14 dpi, all the cattle sera achieved a reactivity plateau. The correlation between the two tests, evaluated by Pearson coefficient, resulted in 0.96. In both tests, the viral strain used for the infection did not influence the reaction profile (Table S1). The detailed results and the comparison with the results previously obtained with the commercial ELISA (ID.vet Capripox DA ELISA) and the VNT by Möller et al. [28] are summarized in Table S1: the results of the four compared tests are consistent, however, it is relevant to highlight that both the two new ELISAs detected seroconversion 1 or 2 weeks earlier in 5 of 18 cattle, compared to either VNT or ID.vet kit. Furthermore, seropositivity was never detected in one animal by either the VNT or ID.vet test, in contrast to both ELISAs which became positive from 14 dpi. The comparison denotes a better sensitivity of the newly developed ELISAs.

### 3.4. Evaluation of Goat Sera in the Competitive and Indirect ELISAs

**Figure 4:**
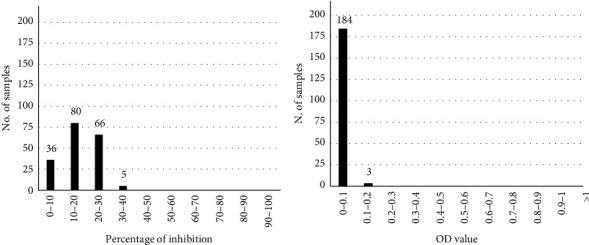
Frequency values distribution obtained examining a population of 187 goatpox-negative goat sera. Results are expressed as percentage of inhibition in the competitive ELISA tests (a) and as optical density (OD) in the indirect ELISA test (b). On the *x*-axis, the percentages of inhibition (a) and the OD value (b) are reported, while on the *y*-axis the number of the analyzed samples is shown.

Testing of 187 goat sera negative for goatpox virus showed a distribution of PI values in competitive ELISA included in a range of 0%–40% PI and net OD values lower than 0.2 OD in indirect ELISA. Therefore, the cutoffs selected for cattle, namely 50% PI for competitive ELISA and 0.2 OD for indirect ELISA, were confirmed suitable also to test goat sera for the evaluation of the performance of the tests in detecting antibodies to the goatpox virus (Figures 4(a) and 4(b)).

In Table 1 the results from competitive and indirect ELISA are reported and compared with those of VNT and the commercial capripox ELISA test (ID.vet Capripox DA ELISA) previously carried out [30]. The tests performed on sera from GTPV experimentally infected goats provided evidence of seroconversion, albeit with less consistency than in cattle and depending on the sampling time after infection. In general, seroconversion was better detected in intranasally inoculated goats which could be sampled up to 28 dpi, in contrast to sera from those intravenously inoculated which were available for a shorter time (10 dpi); expectedly, seroconversion appeared delayed in in-contact animals. Concerning tests performance, competitive ELISA was the best performant, discovering seroconversion in seven out of eight goats; the kinetics showed 10 days as the minimum time for seroconversion detection. Compared to competitive ELISA, the VNT missed two sera (two goats), the indirect ELISA missed four sera (four goats, though some were borderline positive) and the ID.vet kit missed five sera (four goats). The correlation between the two novel tests, evaluated by the Pearson coefficient, resulted in 0.72.

### 3.5. Evaluation of Sheep Sera in Competitive and Indirect ELISAs

**Figure 5:**
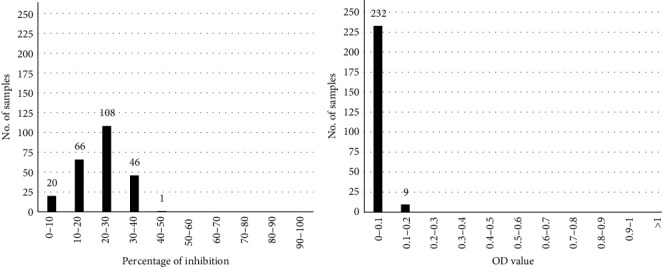
Frequency values distribution obtained examining a population of 241 sheepox-negative sheep sera. Results are expressed as percentage of inhibition in the competitive ELISA test (a) and as optical density (OD) in the indirect ELISA test (b). On the *x*-axis, the percentages of inhibition (a) and the OD value (b) are reported, while on the *y*-axis, the number of the analyzed samples is shown.

Similarily to bovine and goat sera, the evaluation of 241 sheep sera negative for sheeppox showed a PI distribution included in a range of 0%–44% in competitive ELISA and net OD values in indirect ELISA lower than 0.2, with over 95% not exceeding 0.1 (Figures 5(a) and 5(b)). According to these results, the cutoff values of 50% PI for the competitive ELISA and 0.2 OD for indirect ELISA were established also for sheep sera.

Results of testing sera from sheep experimentally infected with two different isolates of SPPV are reported in Table 2, where results of the newly developed ELISAs are compared with those of two further tests (VNT and the commercial Capripox ELISA test–ID.vet) previously carried out [31]. Similarly to what observed in LSDV-infected cattle, both new ELISAs revealed seroconversion within 14 dpi in all the nine sheep (with one borderline value in one animal in indirect ELISA), demonstrating higher sensitivity than VNT and the commercial capripox ELISA kit, that detected only three sheep and two sheep positive at the same time point, respectively. Moreover, positivity was detected uniquely by the competitive ELISA as early as 7 dpi in two sheep.

The correlation between the two new ELISAs, evaluated by the Pearson coefficient, resulted in 0.85. In both tests, no significant difference in reaction profile related to the viral strain used for the infection was observed.

## 4. Discussion

LSD might generate extensive damage to the host's health, as well as a severe economic loss. In fact, LSDV can spread quickly and over long distances, especially when not treated properly. Consequently, the finding of new tools for effective diagnosis and surveillance becomes increasingly important. The final aim of this study was the development of new ELISA assays for LSDV antibody detection, with new formats compared to previously described ELISAs and taking advantage of the use of MAbs. The initial evaluation of the two novel serological assays, using known sera from naïve populations and sera collected sequentially after experimental infections, provided evidence that both ELISAs are feasible, practical and effective for the detection of anti-LSDV antibodies with a high level of specificity and a sensitivity comparatively superior to that shown by the VNT, adopted as gold standard, and by the sole commercial ELISA available.

To this purpose, a panel of 75 MAbs against the virulent LSDV Neethling strain was produced. Through a preliminary characterization, 14 MAbs were selected and further characterized with immunoenzymatic tests. These antibodies reacted in WB with a viral protein of about 35 kDa molecular weight which was effectively recognized also by LSDV positive sera. Subsequently, 4 out of the 14 MAbs were chosen to develop ELISA assays for antibody detection as they targeted an immunogenic epitope, as demonstrated by preliminary competitive ELISA with LSDV positive sera. Moreover, the reciprocal competition between these four MAbs highlighted a similar reactivity profile for MAbs named 3H5 and 2C6, suggesting they bind to similar epitopes, while 2F10 and 2F12 MAbs are likely to target two different epitopes within the same protein. One of the most intensively studied species in the *Poxviridae* family is the *Vaccinia virus* (VV), where the H3L gene codes for a structural membrane protein of about 35 kDa (p35). This protein is one of the most immunogenic and it localizes in the IMV envelope, it binds to heparan sulfate on the cell surface, and it might provide virion attachment to the target cell. The comparative genome analysis of VV with other Poxviruses identified H3L homologous gene also in LSDV [41], namely LSDV-074, and the other capripoxviruses [41] which codify for a genus specific protein of 32 kDa as predicted molecular weight. Thus, our findings suggested that the MAbs selected could target the LSDV-074 gene product; to confirm it, a part of the ongoing research involves the expression of the protein codified by LSDV 074 gene through the *Escherichia coli* system.

The selected MAbs were used to develop two ELISA tests: a competitive ELISA that detected competition between MAbs and serum antibodies for the binding to the viral antigen, regardless of the species, and an indirect ELISA that exploited an anti species labelled antibody recognizing the serum immunoglobulins bound to the antigen. Both tests adopted the strategy to capture the previously inactivated LSDV virus onto the solid phase using a capture MAb precoated onto ELISA microplates. This strategy prevented the need for virus purification which usually requires complex procedures and allow the use of unpurified supernatant of LSDV infected cells as the source of antigen since the virus is immunopurified and concentrated onto the microplate by the selected specific MAb. To design and optimize the competitive ELISA test, all the combinations of the four selected MAbs were evaluated in sandwich ELISA to determine the best MAb pair to properly capture and detect the viral antigen, taking into account that the second MAb (peroxidase-conjugated) has the dual function of binding to the virus as a detector antibody and of competing with antibodies of positive sera for binding to the virus itself. The 2F10 and 2C6 MAbs were, respectively, chosen as the best match of catcher and detector for the competitive ELISA. In the indirect ELISA, the same capture antibody (2F10 MAb) was selected after verification that it properly exposed the viral antigen to the reaction of anti-LSDV antibodies in sera from infected animals.

The specificity of both tests was evaluated with a panel of 192 cattle sera collected in Italy (LSD free country); testing of known LSDV negative sera allowed us to set the cutoff values of the two novel assays, namely 50% percentage inhibition for the competitive ELISA and 0.20 OD for the indirect ELISA, resulting in diagnostic specificity of 100% and 99.5%, respectively. Analysing the sera of 18 cattle experimentally infected with LSDV and sequentially sampled for up to 4 weeks, we had evidence that both assays detected seroconversion from 7 to 14 dpi, regardless of the virus strain used for the infection, and it was observed an almost perfect correlation between the two assays. The cattle sera from experimental infection tested in the present study were previously evaluated [28, 29] with the commercial ID.vet Capripox Double antigen (DA) ELISA and with the VNT; the comparison of results obtained with the four tests provided evidence of improved sensitivity of the newly developed ELISAs, which detected seroconversion earlier in 5 out of 18 cattle.

To evaluate the performance of the tests with sera from animals infected by other capripoxviruses (GTPV and SPPV), a panel of goat and sheep sera experimentally infected was analyzed, after having confirmed the same cutoff values established for cattle by analyzing a similar number of negative animals for each species and obtaining 100% specificity. The correlation coefficient of the two newly developed tests with experimental goat and sheep sera resulted in 0.72 and 0.85, respectively, lower than that observed in cattle sera. The analysis of experimental sheep sera produced similar results to those observed in cattle, with seroconversion detected in all infected animals within 14 dpi by the two new ELISAs, that is earlier in the majority of animals compared to both VNT and the ID.vet Capripox kit, according to previously reported results [31].

The comparison of tests performances in goats experimentally infected with GTPV, and previously analyzed using ID.vet Capripox DA ELISA and VNT [30], indicating the new competitive ELISA as the best performant test, highlighting seroconversion in seven out of eight goats, followed by VNT which detected positivity in five animals, then by indirect ELISA and ID.vet Capripox ELISA which evidenced positivity in only three goats. Substantially, the newly developed ELISAs worked effectively for serological examination of LSDV infection, as well as for GTPV and SPPV infection in goats and sheep, respectively. The results obtained from the capripox infected sera in competitive ELISA suggested the presence of a common immunogenic epitope, recognized by 2C6 MAb. This is consistent with the analysis of the amino acid sequences of the protein codified by the LSDV 074 gene and those of other capripoxviruses used in the present study, which resulted in more than 96% of homology. However, despite the three viruses showing a very high-amino acid homology, the cross-reactivity of the humoral response detected by the indirect ELISA in goats was lower than in sheep. Possibly, the reactivity profiles in indirect ELISA could be influenced by the infection route, and by a different kinetic of the antibody response in goats, correlated with the time of sampling. Indeed, it should also be considered that the competitive ELISA, as well as the VNT, are independent on the immunoglobulins class of antibodies and therefore can detect early and late antibodies (both IgM and IgG), while the indirect ELISA described here uses a secondary antibody consisting of an anti ruminant IgG MAb, thus dependent on the IgG kinetic.

In conclusion, our results highlighted the importance of MAbs as diagnostic tools. By using a purified viral antigen we obtained a large panel of MAbs, however, so far we have characterized only part of them, and the experiments to study the others are still ongoing. It is noteworthy that two of the MAbs produced and characterized in this study have been used to develop a lateral flow device for rapid LSDV antigen detection in field conditions [42].

The selected MAbs allowed us to develop two ELISA assays using a crude viral antigen, which permits to display a broad spectrum of immunogenic epitopes to the positive sera recognition, especially in indirect ELISA, where the serum immunoglobulins G against the specific antigen are detected. Moreover, for the first time, we have described a competitive ELISA test to detect antibodies against capripox viruses, which potentially can detect all antibody classes since it is based on the competition between MAb and serum antibodies for the immunogenic target epitope.

The use of known experimental sera for the validation of new serological assays before using them in field conditions is important to acquire scientific knowledge on the performance and reliability of the tests; on the other hand, the limited accessibility and availability of experimental sera with a “known status” for LSDV and other capripox viruses makes these samples valuable and extremely useful for the validity of our study. Further studies and validation of the new ELISAs also in field conditions will be conducted in order to acquire additional information on their effectiveness for LSD surveillance and control.

## Figures and Tables

**Table 1:** Goat sera collected at different time points after experimental infection with GTPV, strain “V/103.”

Animal ID	Infection route	dpi	VNT (titer)	Capripox DA ELISA (ID.vet) (S/P%)	MAbs-based competitive ELISA PI values	MAbs-based indirect ELISA OD values
Z/253	Intravenous and subcutaneous	0	<1 : 10	−1	0	0
		7	<1 : 10	−1	4	0.035
		10	1 : 13^*∗*^	−1	45	0.105

Z/254	Intravenous and subcutaneous	7	1 : 13^*∗*^	0	37	0.004
		10	1 : 80^*∗*^	16	67^*∗*^	0.034

Z/255	Intravenous and subcutaneous	0	<1 : 10	0	0	0
		7	<1 : 10	0	0	0
		10	1 : 25^*∗*^	53^*∗*^	54^*∗*^	0.153

Z/256	Intranasal	0	<1 : 10	−1	0	0
		7	<1 : 10	−1	0	0.045
		15	1 : 16^*∗*^	23	78^*∗*^	0,39^*∗*^

Z/257	Intranasal	0	<1 : 10	−1	0	0
		7	<1 : 10	−1	0	0.008
		28	1 : 80^*∗*^	83^*∗*^	93^*∗*^	0.856^*∗*^

Z/258	Intranasal	0	<1 : 10	−1	0	0.132
		7	<1 : 10	−1	0	0.106
		15	1 : 80^*∗*^	1	81^*∗*^	0.727^*∗*^
		28	1 : 128^*∗*^	65^*∗*^	94^*∗*^	1.142^*∗*^

Z/259	In contact	0	<1 : 10	6	0	0.018
		7	<1 : 10	1	0	0.01
		15	<1 : 10	−1	0	0.006
		21	<1 : 10	−1	34	0.023
		23	1 : 40^*∗*^	0	75^*∗*^	0.109

Z/260	In contact	0	<1 : 10	−1	0	0
		7	<1 : 10	−1	0	0.005
		15	<1 : 10	−1	0	0.003
		21	<1 : 10	0	0	0
		28	1 : 13^*∗*^	15	51^*∗*^	0.145

The infection route and the results of the virus neutralization test (VNT), the ID.vet Capripox DA ELISA test (S/P%), and the new competitive and indirect ELISAs based on monoclonal antibodies are reported. The positive results are highlighted with the  ^*∗*^ symbol, according to the cutoff value established for each test.

**Table 2:** Sheep sera collected at different time points after experimental infection with two strains of SPPV.

Challenge virus	Animal ID	Infection route	dpi	VNT (titer)	Capripox DA ELISA (ID.vet) (S/P%)	MAbs-based competitive ELISA (PI values)	MAbs-based Indirect ELISA (OD values)
SPPV-Egypt/2018	S-01	Intravenous	0	<1 : 10	0	0	0
			7	<1 : 10	15	51^*∗*^	0
			14	1 : 20^*∗*^	31^*∗*^	77^*∗*^	0.185
			21	1 : 160^*∗*^	57^*∗*^	93^*∗*^	0.711^*∗*^
			28	1 : 160^*∗*^	87^*∗*^	93^*∗*^	0.736^*∗*^

SPPV-Egypt/2018	S-02	Intravenous	0	<1 : 10	0	0	0.018
			7	<1 : 10	61^*∗*^	45	0.012
			14	1 : 13	22	66^*∗*^	0.407^*∗*^

SPPV-Egypt/2018	S-03	Intravenous	0	<1 : 10	1	0	0
			7	<1 : 10	9	51^*∗*^	0,03
			14	1 : 16^*∗*^	23	80^*∗*^	0.476^*∗*^
			21	1 : 80^*∗*^	98^*∗*^	95^*∗*^	0.653^*∗*^
			28	1 : 200^*∗*^	134^*∗*^	93^*∗*^	0.753^*∗*^

SPPV-Egypt/2018	S-04	Intranasal	0	<1 : 10	0	0	0.022
			7	<1 : 10	0	0	0
			14	<1 : 10	5	60^*∗*^	0.413^*∗*^

SPPV-Egypt/2018	S-05	Intranasal	0	<1 : 10	0	0	0.099
			7	<1 : 10	1	2	0.088
			14	1 : 13	25	67^*∗*^	0.503^*∗*^

SPPV-Egypt/2018	S-06	Intranasal	0	<1 : 10	0	0	0
			7	<1 : 10	2	29	0
			14	1 : 16^*∗*^	12	52^*∗*^	0.224^*∗*^

SPPV-India/2013	S-09	Intravenous	0	<1 : 10	0	0	0.009
			7	<1 : 10	21	37	0
			14	1 : 13	16	67^*∗*^	0.471^*∗*^

SPPV-India/2013	S-12	Intranasal	0	<1 : 10	1	0	0
			7	<1 : 10	0	0	0
			14	<1 : 10	36^*∗*^	65^*∗*^	0.251^*∗*^

SPPV-India/2013	S-14	Intranasal	0	<1 : 10	−1	0	0
			7	<1 : 10	−1	3	0
			14	<1 : 10	10	64^*∗*^	0.327^*∗*^
			21	1 : 40^*∗*^	85^*∗*^	95^*∗*^	0.756^*∗*^
			28	1 : 256^*∗*^	136^*∗*^	96^*∗*^	0.792^*∗*^

The virus used for the infection and the infection route are reported, together with results of the virus neutralization test (VNT), the ID.vet Capripox DA ELISA test (S/P%), and the new competitive and indirect ELISAs based on monoclonal antibodies. The positive results are highlighted with the  ^*∗*^ symbol according to the cutoff value established for each test.

**Table 3:** Isotype and reactivity profile of the 14 MAbs reactive in western blotting against a 35 kDa viral protein.

Monoclonal antibody	Indirect ELISA	MAbs reciprocal competition			
	Neethling LSDV (OD value)	PI vs. 3H5-PO	PI vs. 2C6-PO	PI vs 2F10-PO	Isotype
2C6	2.0	96^*∗*^	97^*∗*^	96^*∗*^	IgG1
3H5	1.9	96^*∗*^	96^*∗*^	54^+^	IgG1
5C8	1.5	60^+^	64^+^	53^+^	IgG2a
2F10	2.0	37	40	96^*∗*^	IgG1
7B7	1.8	31	31	93^*∗*^	IgG1
4E8	1.6	26	36	92^*∗*^	IgG2a
6G4	2.1	28	35	91^*∗*^	IgG1
2A9	2.1	24	14	88^*∗*^	IgG1
2A6	2.2	22	17	70^*∗*^	IgG1
2C9	2.1	21	17	66^+^	IgG1
2F12	2.0	20	19	58^+^	IgG1
8C4	1.8	13	27	52^+^	IgG1
3D12	2.1	1	11	34	IgG1
2E11	1.9	12	12	30	IgG1

The table presents the OD values of indirect ELISA obtained by each MAb against the Neethling strain, and the percentage of inhibition (PI) of competitive ELISA between the tested MAbs (competitor) and three peroxidase- (PO-) conjugated MAbs. High-competition value is shown with  ^*∗*^ symbol, partial competition is indicated with + symbol, and the numbers with no symbol show low or no competition.

## Data Availability

All the data are present in the manuscript or in the supplementary material.

## Supplementary Materials

Supplementary Materials The supplementary material file (Table S1) presents all the cattle sera from the experimental infection that were collected weekly up to 28 dpi and were analyzed with ID.vet Capripox DA ELISA, VNT, and both the new ELISAs based on MAbs.
