# Supplementary material for: ELISA Methods Based on Monoclonal Antibodies for the Serological Diagnosis of Lumpy Skin Disease
Source: Transbound Emerg Dis. 2023 Jul 27;2023:8378153. doi: 10.1155/2023/8378153 (PMC12017155; doi:10.1155/2023/8378153)
Supplement: Supplementary Materials — The supplementary material file (Table S1) presents all the cattle sera from the experimental infection that were collected weekly up to 28 dpi and were analyzed with ID.vet Capripox DA ELISA, VNT, and both the new ELISAs based on MAbs. [file 8378153.f1.pdf]

Table S1: Cattle sera collected weekly up to 28 days post experimental infection with LSDV. The virus used for the infection of each animal and results of VNT, ID.vet Capripox DA ELISA, and the new competitive and indirect ELISAs based on monoclonal antibodies are reported. The positive results are highlighted with the * symbol, according to the cut-off value established for each test.

**Inoculated virus**	**Animal ID**	**dpi**	**VNT (titer)**	**Capripox DA ELISA (ID.vet) (S/P%)**	**MAbs-based Competitive ELISA (PI)**	**MAbs-based Indirect ELISA (OD)**
LSDV vaccine strain “Neethling”	124	-2	<1:10	13	0	0,018
		7	<1:10	4	0	0,073
		14	1:18*	99*	84*	1,248*
		21	1:57*	168*	94*	1,418*
		28	1:45*	196*	95*	1,525*
LSDV vaccine strain “Neethling”	128	-2	<1:10	-4	0	0
		7	<1:10	9	0	0
		14	1:7	-1	66*	0,810*
		21	1:7	-6	69*	0,889*
		28	1:7	22	73*	0,965*
LSDV vaccine strain “Neethling”	129	-2	<1:10	-1	0	0
		7	<1:10	10	0	0,033
		14	1:18*	34*	78*	0,552*
		21	1:45*	49*	88*	0,892*
		28	1:71*	101*	94*	1,206*
LSDV vaccine strain “Neethling”	133	-2	<1:10	3	0	0
		7	<1:10	16	4	0,027
		14	1:13*	37*	83*	1,252*
		21	1:45*	36*	85*	1,515*
		28	1:180*	129*	90*	1,574*
LSDV vaccine strain “Neethling”	193	-2	<1:10	4	0	0,089
		7	<1:10	14	0	0,043
		14	<1:10	17	53*	0,812*
		21	<1:10	43*	62*	0,919*
		28	1:7	36*	65*	0,895*
LSDV vaccine strain “Neethling”	195	-2	<1:10	-1	0	0
		7	<1:10	12	0	0
		14	<1:10	38*	71*	0,677*
		21	<1:10	50*	80*	1,15*
		28	1:13*	88*	81*	1,038*
LSDV North Macedonia 2016	200	-2	<1:10	3	0	0,046
		7	<1:10	13	0	0,007
		14	1:28*	71*	79*	1,068*
		21	1:28*	169*	87*	1,308*
		28	1:90*	196*	91*	1,480*
LSDV North Macedonia 2016	285	-2	<1:10	7	0	0
		7	<1:10	20	0	0,037
		14	1:28*	42*	88*	1,212*
		21	1:57*	65*	92*	1,492*
		28	1:90*	136*	94*	1,517*
LSDV North Macedonia 2016	286	-2	<1:10	13	0	0,168
		7	<1:10	0	0	0,097
		14	1:14*	-3	60*	1,069*
		21	1:36*	12	75*	1,165*
		28	1:45*	71*	87*	1,46*
LSDV North Macedonia 2016	477	0	<1:10	-1	0	0
		7	<1:10	-2	0	0,008
		14	1:100*	10	68*	0,871*
		21	1:200*	69*	80*	1,206*
		28	1:400*	190*	88*	1,398*
LSDV North Macedonia 2016	484	0	<1:10	0	1	0,233
		7	<1:10	0	6	0,166
		14	1:128*	89*	76*	1,355*
		21	1:200*	130*	81*	1,254*
		28	1:200*	166*	88*	1,407*

LSDV North Macedonia 2016	487	0	<1:10	-1	0	0,01
		7	<1:10	-2	0	0
		14	1:25*	18	63*	0,680*
		21	1:400*	112*	89*	1,288*
		28	1:1280*	128*	96*	1,605*
LSDV North Macedonia 2016	544	-2	<1:10	-1	0	0
		7	<1:10	-2	0	0,073
		14	1:80*	24	74*	1,292*
		21	1:512*	64*	89*	1,334*
		28	1:320*	144*	94*	1,493*
LSDV North Macedonia 2016	556	-2	<1:10	0	0	0
		7	<1:10	-2	0	0,016
		14	1:64*	94*	78*	1,209*
		21	1:200*	86*	89*	1,443*
		28	1:320*	208*	95*	1,571*
LSDV Nigeria 2019	R-47	-2	<1:10	0	0	0
		7	<1:10	-1	6	0
		14	<1:10	2	51*	0,658*
		21	1:13*	43*	93*	0,994*
		28	1:80*	153*	96*	1,114*
LSDV Nigeria 2019	R-81	-2	<1:10	0	0	0
		7	<1:10	-2	0	0
		14	1:13*	10	66*	0,778*
		21	1:80*	50*	88*	0,967*
		28	1:100*	127*	84*	1,119*
LSDV Nigeria 2019	R-83	-2	<1:10	-1	15	0
		7	<1:10	-1	3	0,004
		14	<1:10	13	53*	0,459*
		21	1:20*	89*	94*	1,141*
		28	1:50*	125*	95*	1,052*
LSDV Nigeria 2019	R-84	-2	<1:10	-1	14	0
		7	<1:10	-1	10	0
		14	<1:10	17	76*	0,662*
		21	1:80*	92*	92*	1,045*
		28	1:64*	135*	93*	1,031*
